# Scanning Electrochemical Microscopy of Nystatin-Treated Yeast Used for Biofuel Cells

**DOI:** 10.3390/s26020605

**Published:** 2026-01-16

**Authors:** Katazyna Blazevic, Antanas Zinovicius, Juste Rozene, Tomas Mockaitis, Ingrida Bruzaite, Laisvidas Striska, Evaldas Balciunas, Arunas Ramanavicius, Almira Ramanaviciene, Inga Morkvenaite

**Affiliations:** 1 NanoTechnas—Center of Nanotechnology and Materials Science, Institute of Chemistry, Faculty of Chemistry and Geosciences, Vilnius University, Naugarduko St. 24, LT-03225 Vilnius, Lithuania; katazyna.blazevic@chgf.vu.lt; 2 Department of Nanotechnology, State Research Institute Centre for Physical Sciences and Technology, Sauletekio Ave. 3, LT-10257 Vilnius, Lithuania; antanas.zinovicius@ftmc.lt (A.Z.); tomas.mockaitis@ftmc.lt (T.M.); ingrida.bruzaite@vilniustech.lt (I.B.); laisvidas.striska@ftmc.lt (L.S.); evaldas.balciunas@ftmc.lt (E.B.); 3 Department of Mechatronics, Robotics, and Digital Manufacturing, Vilnius Gediminas Technical University, Plytines St. 25, LT-10105 Vilnius, Lithuania; juste.rozene@vilniustech.lt; 4 Department of Chemistry and Bioengineering, Faculty of Fundamental Sciences, Vilnius Gediminas Technical University, Sauletekio Ave. 11, LT-10223 Vilnius, Lithuania; 5 Department of Electrical Engineering, Vilnius Gediminas Technical University, Plytines St. 25, LT-10105 Vilnius, Lithuania; 6 Department of Physical Chemistry, Faculty of Chemistry and Geosciences, Vilnius University, Naugarduko St. 24, LT-03225 Vilnius, Lithuania; arunas.ramanavicius@chf.vu.lt

**Keywords:** *Saccharomyces cerevisiae*, microbial fuel cell, biofuel cells, nystatin, SECM

## Abstract

Biofuel cells (BFCs) generate electricity by converting chemical energy into electrical energy using biological systems. *Saccharomyces cerevisiae* (yeast) is an attractive biocatalyst for BFCs due to its robustness, low cost, and metabolic versatility; however, electron transfer from the intracellular reactions to the electrode is limited by the cell membrane. Nystatin is an antifungal antibiotic that increases the permeability of fungal membranes. We hypothesized that sub-lethal nystatin treatment could enhance mediator-assisted electron transfer without compromising cell viability. In this work, yeast was treated with nystatin during cultivation at concentrations of up to 6 µg/mL and combined with a dual-mediator system consisting of a lipophilic mediator (9,10-phenanthrenequinone, PQ) and a hydrophilic mediator (potassium ferricyanide). Scanning electrochemical microscopy revealed that the dual-mediator system increased local current responses by approximately fivefold compared to a single mediator (from ~11 pA to ~59 pA), and that nystatin-treated yeast exhibited higher local electrochemical activity than untreated yeast (maximum currents of ~0.476 nA versus ~0.303 nA). Microbial fuel cell measurements showed that nystatin treatment increased the maximum power density from approximately 0.58 mW/m^2^ to approximately 0.62 mW/m^2^ under identical conditions. Nystatin concentrations between 4 and 5 µg/mL maintain yeast viability at near-control levels, while higher concentrations cause a decrease in viability. These results demonstrate that controlled, sub-lethal membrane permeabilization combined with a dual-mediator strategy can enhance electron transfer in yeast-based biofuel cells.

## 1. Introduction

A biofuel cell (BFC) is a device that converts chemical energy into electrical energy using biological systems and their intrinsic electrochemical functionalities [1,2,3]. As a bioelectrochemical system, it can employ whole cells, such as unicellular fungi, or enzymes. Usually, it consists of two electrodes—an anode and a cathode—which are separated by a semipermeable membrane in an electrolyte solution. Cells (like unicellular fungi) or enzymes are placed in the solution of the anode compartment or immobilized onto the anode itself [4]. In the presence of a suitable substrate, such as glucose, oxidation occurs at the anode, generating electrons that travel to the cathode, where oxygen is reduced to water [5].

In this work, we focus solely on microbial fuel cells (MFCs), where electric current is produced by microbial cells. The yeast *Saccharomyces cerevisiae* is often used in research because of its simplicity and low cost [6,7,8]. The National Aeronautics and Space Administration used *S. cerevisiae* in space missions because this yeast species can survive long periods with minimal viability support, enabling long-duration, self-contained biosensing [9]. In these biosensors, yeast serves as the biorecognition element, and a transducer converts its metabolic or stress responses into measurable signals. Optical or electrochemical readouts quantify radiation effects, toxicity, dissolved oxygen, and biochemical oxygen demand (BOD), with mediator-assisted electron transfer enhancing stability and sensitivity. Beyond sensing, researchers have successfully applied yeast-based MFCs for electricity generation from wastewater [10].

One problem that prevents widespread industrial MFC application is the low electron transfer capability of cellular membranes and walls [11]. Redox mediators improve electron transfer, transferring the charge to the electrode [12,13]. Redox couples, such as oxidized/reduced forms of enzymes, glutathione, NAD^+^/NADH, or NADP^+^/NADPH, interact with redox mediators [14,15]. Redox mediators can be divided into lipophilic and hydrophilic depending on their chemical structure, and can be used as single or double mediator systems in BFC [16,17].

One of the redox mediators used in *S. cerevisiae*-based MFCs is potassium ferricyanide (K_3_[Fe(CN)_6_]). It is a hydrophilic mediator with stable Fe(II) and Fe(III) redox states [18,19]. 9,10-phenanthrenequinone (PQ) has been shown to work as a lipophilic redox mediator [19,20,21]. Upon interaction with the yeast cell membrane, PQ transfers electrons from the cell interior to the cell exterior and passes charge to K_3_[Fe(CN)_6_]. The transfer of electrons from the cells inside to the electrode occurs through two redox mediators interacting with each other, with lipophilic mediators inside the cell and hydrophilic mediators outside the cell [22,23].

Nystatin (Nys) is an antifungal antibiotic whose principle of action is based on forming pores in the membranes of fungal cells [24,25,26]. Based on its structure, nystatin belongs to polyene antimycotics, other examples of which include amphotericin B [27]. Nystatin is selective for ergosterol in fungal cell membranes because it binds only to ergosterol due to the additional double bonds in ergosterol, which facilitate attachment to polyenes. Therefore, channels are formed in fungal cell membranes. Meanwhile, it causes low toxicity to human cells, as, instead of ergosterol, the mammalian cell membrane has cholesterol [28]. Thus, this channel creates an electrochemical potential gradient through which K^+^ ions can travel freely to (or from) the cell [29]. At high concentrations of Nys, fungal variants have numerous pores that allow the release of intracellular fluid or the formation of excess K^+^ ions, leading to cell death–apoptosis [30].

From a bioelectrochemical perspective, increased membrane permeability is expected to facilitate electron transfer in mediator-assisted MFCs by reducing mass-transport limitations at the cell membrane. In yeast-based systems, intracellular reducing equivalents such as NADH and reduced glutathione are spatially separated from extracellular hydrophilic mediators by the plasma membrane and cell wall. The formation of nystatin-induced pores may lower this barrier, enabling more efficient interaction between intracellular redox species, lipophilic mediators embedded in the membrane, and hydrophilic mediators in the extracellular phase. At sub-lethal concentrations, such controlled permeabilization has the potential to enhance electron flux toward the electrode without significantly impairing metabolic activity.

To our knowledge, this is the first study to demonstrate that controlled, sub-lethal antifungal-induced membrane permeabilization (using nystatin) can be exploited to enhance mediator-assisted electron transfer in yeast-based BMCs and to visualize this enhancement directly using scanning electrochemical microscopy. While previous studies have been focused on mediator chemistry or electrode modification, the present work targets the biological membrane barrier itself as a tunable element in bioelectrochemical systems.

Based on this rationale, we hypothesized that low concentrations of nystatin could enhance mediator-mediated electron transport in *Saccharomyces cerevisiae*, resulting in increased electrochemical activity and higher power output in microbial fuel cells.

In this article, *S. cerevisiae*-based MFCs were investigated. One- and two-electron transfer mediators and nystatin were chosen for testing to create a convenient system and an efficient MFC. The potential application of small concentrations of nystatin to improve charge transfer across yeast cell membranes by pore formation, thereby increasing the efficiency of the MFC. Scanning electrochemical microscopy (SECM) has been chosen to visualize the electrochemical activity produced by the yeast. Studies on the power output of the developed MFC, which were performed by varying the external loads, were also conducted.

## 2. Materials and Methods

Dry yeast (*Saccharomyces cerevisiae*) was purchased from Dr. Oetker Baltic (Vilnius, Lithuania). Potassium ferricyanide (K_3_[Fe(CN)_6_]) (>99%), nystatin dihydrate (C_47_H_71_NO_15_·2H_2_O) (Nys) (85%) were purchased from Carl Roth (Karlsruhe, Germany). Yeast extract peptone dextrose (YPD), 9,10-phenanthrenequinone (PQ), poly-L-lysine, 0.01%, D-(+)-glucose (C_6_H_12_O_6_) (99%), potassium chloride (KCl) (99%), monosodium phosphate (NaH_2_PO_4_) (99%), disodium hydrogen phosphate dodecahydrate (Na_2_HPO_4_·12H_2_O) (99%), and sodium acetate (CH_3_COONa) (99%) were purchased from Merck (Darmstadt, Germany). A disk-shaped platinum ultramicroelectrode (UME) probe with 5 μm radius was purchased from Sensolytics (Bochum, Germany). A graphite rod and a counter platinum electrode were purchased from Metrohm AG (Herisau, Switzerland). The 0.5 M phosphate-acetate buffered solution (PBS-A, pH 6.8) was prepared by dissolving 0.05 M CH_3_COONa, 0.05 M NaH_2_PO_4_, 0.05 M Na_2_HPO_4_, and 0.1 M KCl in distilled water. 0.1 M PBS solution was prepared in an analogous way.

### 2.1. Cell Cultivation and Nystatin-Treatment

0.2 g of dry yeast was mixed with 1 g of YPD in an Erlenmeyer flask containing 20 mL of distilled water. In the case of nystatin treatment, up to 6 μg/mL of nystatin was added to the media. Each flask was then shaken for 24 h at room temperature to achieve a homogenous culture.

The resulting yeast cultures were then centrifuged for 3 min at 3× *g*. After discarding the supernatant, the cell pellet was washed three times with PBS-A. The wet yeast mass was finally diluted to a 1 g/mL suspension in PBS-A.

2 µL of the working yeast suspension was immobilized on the anode in the MFC or on the working electrode in the three-electrode electrochemical cell.

### 2.2. Cyclic Voltammetry

The electrochemical characterization of the MFCs was conducted using a potentiostat/galvanostat PGSTAT 30 (Autolab, Utrecht, The Netherlands) controlled by NOVA 2.1 software via cyclic voltammetry (CV). A three-electrode system was employed for the measurements. A 3 mm diameter graphite rod electrode (Sigma–Aldrich, Steinheim, Germany) served as the working electrode, and an Ag/AgCl (3 M KCl) electrode (Metrohm AG, Herisau, Switzerland) was used as the reference electrode. A platinum electrode (Metrohm AG, Herisau, Switzerland) acted as the counter electrode.

All experiments were performed at room temperature in borosilicate glass titration vessels under constant stirring. The electrochemical cell was housed in a Faraday cage with an earth terminal (Autolab, Utrecht, The Netherlands).

Cyclic voltammograms were recorded at a scan rate of 0.1 V/s with a potential step of 0.01 V over a range from −0.6 V to +0.8 V. Three cycles of cyclic voltammetry were performed, and the data from the final cycle were plotted. The main parameters are summarized in Table 1.

Higher mediator concentrations were used for CV to obtain well-resolved redox peaks. In contrast, lower concentrations were used for SECM and MFC measurements to minimize mass-transport limitations and mediator-induced biological perturbations.

### 2.3. Scanning Electrochemical Microscopy

**Figure 1:**
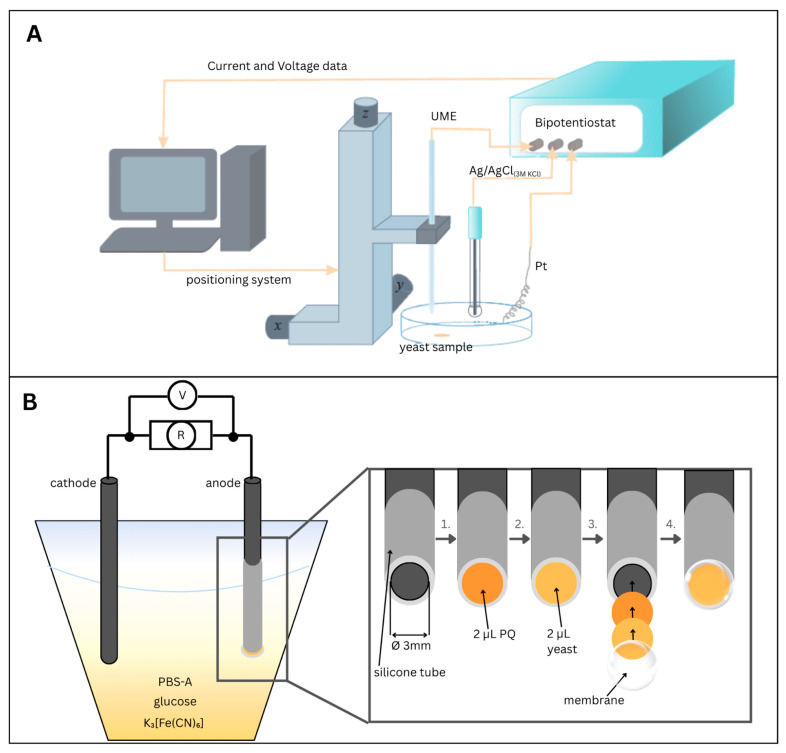
(**A**)—Schematic of a scanning electrochemical microscope. (**B**)—Schematic of microbial fuel cell-generated power test equipment with schematic representation of the anode preparation process: 1. Deposition of 2 μL of lipophilic redox mediator (PQ) onto the surface of a 3 mm diameter graphite rod electrode; 2. Addition of 2 μL yeast suspension on top of the dried PQ layer; 3. Illustration of both immobilized layers (PQ and yeast) and subsequent coverage of the modified electrode with a semi-permeable membrane; 4. Final configuration of the fully assembled electrode, featuring immobilized components and a membrane covering the anode.

The scanning electrochemical microscope (Sensolytics, Bochum, Germany) consists of a three-electrode system (Figure 1A): a working platinum disc-shaped UME with a 5 µm radius, a reference Ag/AgCl_(3M KCl)_, and a counter–platinum electrode. The positioning system allows the UME to move in 3D space. Horizontal scans are performed in generation-collection (GC-SECM) mode at a 20 μm distance from the surface and a potential of +400 mV. The horizontal scans were recorded in 6-min intervals in the presence of hydrophilic (0.6 mM potassium ferricyanide (K_3_[Fe(CN)_6_])) and lipophilic (0.04 mM PQ (9,10-phenanthrenequinone)) redox mediators. The main parameters are summarized in Table 1.

Sample preparation: Poly-L-lysine 0.01% (10 μL) was first added to a clean Petri dish. When the droplet is completely dry, a drop (2 μL) of the nystatin-treated or untreated yeast suspension is immobilized.

### 2.4. Microbial Fuel Cell Power Measurements

Our MFC consists of two (graphite rod) electrodes (Figure 1B), where the anode is the working graphite electrode with a 3 mm diameter. The anode was first coated with 2 μL of a 3 mM PQ solution and dried. Then, 2 μL of yeast suspension was immobilized, covering the anode with a semipermeable membrane. After immobilization of PQ and yeast, the anode was covered with a semi-permeable membrane with a 3 µm pore size. This membrane physically retained the yeast cells on the electrode surface while allowing the diffusion of small molecules, such as glucose, oxygen, and redox mediators. The membrane, therefore, functioned as a mechanical immobilization layer rather than as an ion-exchange separator. The cathode is the counter bare graphite electrode immersed in an electrolyte containing PBS-A, yeast, glucose (60 mM), and the redox mediator K_3_[Fe(CN)_6_] (0.6 mM). The cathode was operated under ambient laboratory conditions, with dissolved oxygen from air acting as the terminal electron acceptor for the cathodic reaction. The main parameters are summarized in Table 1.

MFC efficiency measurements start with an open-circuit measurement. Then the resistance (2.3 MΩ, 1 MΩ, 500 kΩ, 50 kΩ, 1 kΩ, 100 Ω) is applied incrementally, and the generated voltage is measured for each resistance value.

### 2.5. Calculations

Electrochemical measurement data were analyzed using the Hill function, which describes the relationship between the current (I) and the concentration (C) of the selected substrate, either K_3_[Fe(CN)_6_] or PQ, leading to:

$$I=\frac{I_{max}*C^{n}}{k^{n}+C^{n}}$$

where *I* is the current, C is the substrate concentration, k is a constant corresponding to the substrate concentration at which half of the maximum current response is observed, and n is the Hill coefficient, which characterizes the cooperativity of the interaction between the electrode and the electroactive species.

## 3. Results and Discussion

### 3.1. Nystatin Influence on Yeast Cell Viability

**Figure 2:**
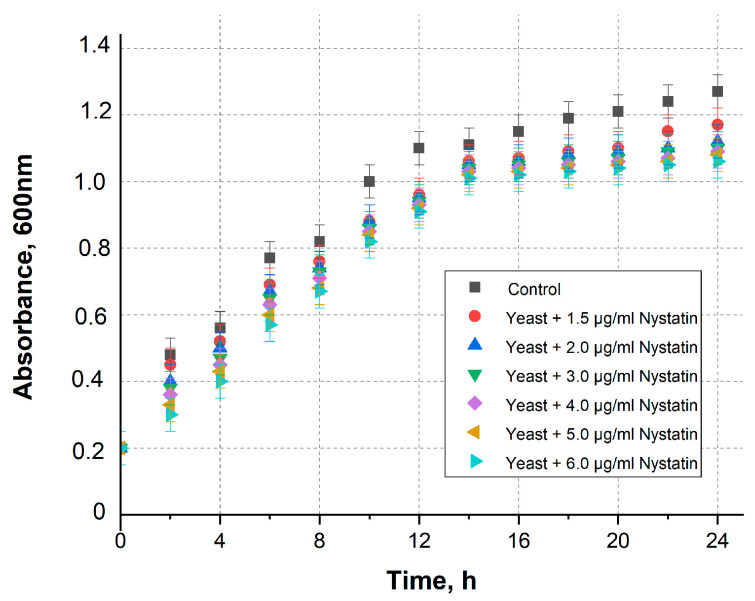
The optical density of yeast culture with various concentrations of nystatin over time +/− standard deviation. Nystatin-untreated cells in YPD were used as a control.

The concentrations of nystatin were selected based on the threshold for apoptosis [31]. A yeast cell viability study was conducted using different nystatin concentrations (Figure 2). A gradual decrease in yeast viability was observed shortly after exposure to nystatin; however, this effect became statistically significant after approximately 12 h of incubation.

These results indicate that nystatin concentrations up to approximately 4–5 µg/mL are sub-lethal and preserve yeast viability, while higher concentrations (≥6 µg/mL) cause a pronounced decrease in viability consistent with the onset of apoptosis. Consequently, the electrochemical effects observed in this work are attributed to controlled membrane permeabilization at sub-lethal nystatin levels rather than to cytotoxic damage.

### 3.2. The Influence of Nystatin on the Efficiency of Electrochemical Microbial Fuel Cells

Cyclic voltammograms were recorded in 0.1 M PBS, pH 6.8, in the presence of 20 mM of K_3_[Fe(CN)_6_] and 100 mM of glucose. The concentration of nystatin was 4.5 µg/mL during yeast cultivation. 2 µL of 3 mM PQ was immobilized on the working electrode surface.

**Figure 3:**
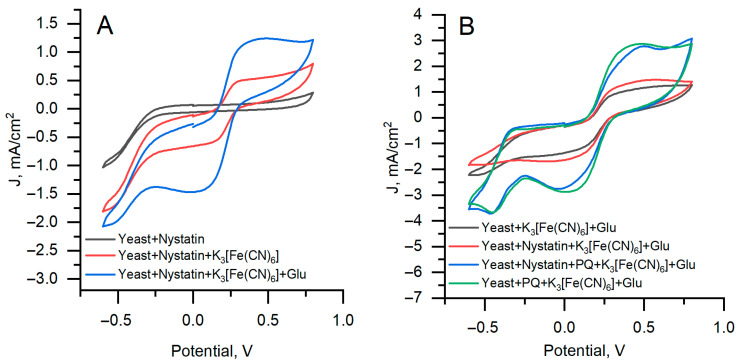
Cyclic voltammogram results for the graphite rod electrode with yeast. (**A**)—Nystatin-treated yeast. (**B**)—Comparison of nystatin-treated and untreated yeast. A potential scan rate of 0.1 V s^−1^ and a potential step of 0.01 V were applied.

The addition of PQ as a lipophilic mediator enables electron transfer from intracellular redox species to the extracellular mediator and electrode, resulting in significantly increased local electrochemical activity, as observed by SECM, and enhanced power output in the microbial fuel cell. Therefore, PQ plays a key functional role in mediating transmembrane electron transfer in this system. The registered cyclic voltammograms are presented in Figure 3.

Nystatin-treated yeast without mediator and glucose (Figure 3A, gray curve) produces a low current response without a well-defined redox couple, consistent with weak endogenous redox activity. The addition of K_3_[Fe(CN)_6_] (Figure 3A, red curve) generates a larger anodic (0.5 mA/cm^2^) and cathodic (−1.6 mA/cm^2^) peak currents. With glucose present (Figure 3A, blue curve), the anodic current at +0.45 V is approximately twice that of the red curve and reaches 1.25 mA/cm^2^. Using a single mediator (K_3_[Fe(CN)_6_]), both untreated and nystatin-treated yeasts reach approximately 1.4 mA/cm^2^ (Figure 3B gray and red curves) with cathodic currents of 4.5 and 5.0 mA/cm^2^, respectively. Adding PQ increased the anodic current to 2.97 mA/cm^2^ and shifted the cathodic peaks to *E*_pc_ 0.15 V with i_pc_ values of 3.0 and 3.1 mA/cm^2^ (Figure 3B blue and green curves).

The results demonstrate that treating yeast cells with nystatin does not significantly impact electric current generation in microbial fuel cells. The difference in current output between nystatin-treated and untreated yeast cells is insignificant, indicating that nystatin does not significantly alter the overall electrochemical response detectable by cyclic voltammetry.

### 3.3. The Scanning Electrochemical Microscopy Studies

**Figure 4:**
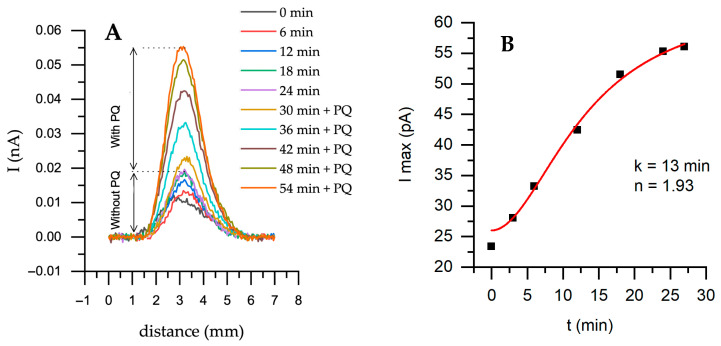
Horizontal scanning by SECM of an untreated yeast sample at different time points with the addition of the electron transfer mediator PQ—0.04 mM; (**A**)—Forward scanning with a step of 50 μm at +400 mV potential; (**B**)—Maximum current from (**A**) part with PQ. Solution: PBS-A in the presence of 0.6 mM K_3_[Fe(CN)_6_]. Fitting was performed using Hill’s equation—nystatin-treated yeast during cultivation.

First, horizontal scans were performed over the untreated yeast sample using one and two redox mediators. Measurements with a single mediator, K_3_[Fe(CN)_6_], were performed for up to 24 min, and then the secondary mediator PQ was added (Figure 4). This represents a single representative time-resolved SECM measurement illustrating the dynamic response following the addition of PQ.

The current has increased during horizontal scanning over a yeast sample with a two-mediator system compared to a one-mediator system (Figure 4A). After adding PQ, the maximum current increased from 19.6 to 55.4 pA (Figure 4B). The obtained data were fitted to Hill’s equation. The k time of 13 min is the time it takes to reach half of the maximum current. The entire process can be divided into three stages: the initial stage (0–5 min), during which the current starts to increase from 25 nA, indicating that PQ slowly participates in intracellular reactions. The second stage is growth (5–20 min). Current increases rapidly and follows the sigmoidal trend, and the system is reaching a positive, cooperative response, also indicated by n (1.93)—the Hill coefficient is close to 2. This is likely due to the accumulation of electroactive species near the electrode surface. Finally, the saturation phase (20–30 min) is when the system reaches its maximum current. This suggests that PQ has been fully incorporated into intracellular reactions and has reached equilibrium.

**Figure 5:**
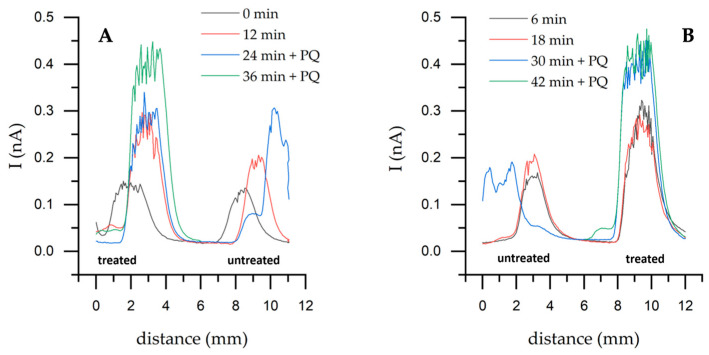
Horizontal SECM scanning over untreated and nystatin-treated yeast samples using a two-mediator system and 1.5 μg/mL of nystatin. Directions are given in SECM’s Cartesian coordinates (*x*, *y*, *z*): scan direction toward +y (**A**); toward −y (**B**). Step 100 μm, speed 100 μm/s, potential + 400 mV. It was scanned in the x and y axes for 5 mm each.

Horizontal scans performed on untreated and nystatin-treated yeast (Figure 5) showed that the current increased with the nystatin-treated yeast. Higher values were achieved with nystatin-treated yeast, reaching 0.476 nA, whereas the highest value with untreated yeast was 0.303 nA. When a single mediator was used, a significant difference of 87 pA was observed between untreated and treated cells at 12 min.

While cyclic voltammetry reflects the overall electrochemical response of the entire electrode surface, SECM provides localized insight into membrane-level electron transfer processes. The enhanced currents observed in SECM and power measurements, therefore, indicate that nystatin primarily improves local electron transport efficiency rather than overall redox kinetics, as detected by CV.

**Figure 6:**
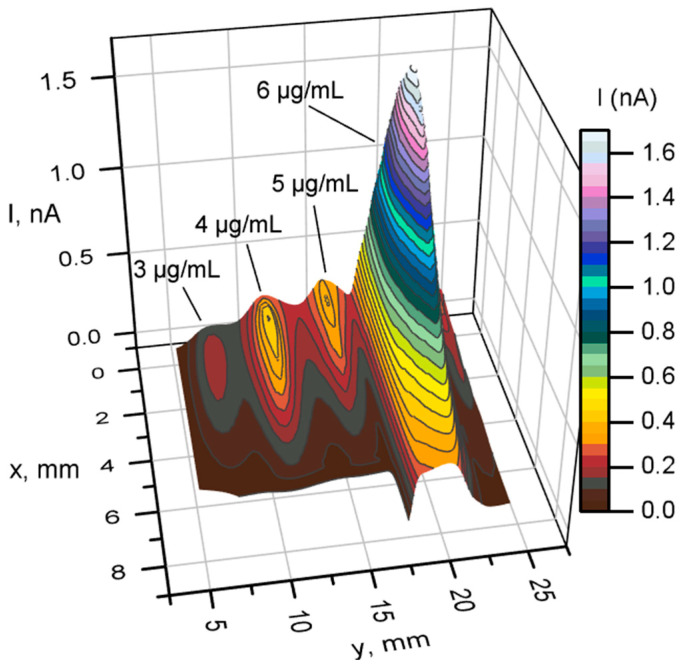
SECM 3D scanning over yeast treated with nystatin using 3, 4, 5, and 6 µg/mL concentrations. Measurements were performed using a two-electron mediator system. Step 200 μm, speed 200 μm/s, potential +400 mV. Nystatin is added during yeast cultivation.

The effects of nystatin concentration on the electrochemical response of the samples were measured using SECM. Figure 6 presents the results obtained by analyzing 4 yeast samples treated with nystatin at 3, 4, 5, and 6 μg/mL concentrations in a Petri dish.

The results show that at 4 μg/mL, the maximum current reached around 0.6 nA (Figure 6). Three-dimensional scan additionally indicates the quality of the immobilized samples. Another topographic scan was performed on yeast treated with 6 μg/mL nystatin.

The results presented in Figure 6 show that increasing the concentration of nystatin in the presence of a two-mediator system increases the generated current over the sample. The highest current of 1.7 nA was achieved at a concentration of 6 μg/mL of nystatin. This suggests that increasing nystatin during the incubation of yeast improves membrane permeability. All obtained topographical scans over the samples show different redox activity. Because yeast cells are living organisms, their activity can vary depending on the growth phase, pH differences, temperature, and other factors. For this reason, general trends are examined.

Summarizing the obtained data, it was found that in the presence of a two-mediator system, nystatin-treated yeast generates a higher current compared to untreated yeast or a single-mediator system. A trend of direct proportionality was also observed at higher nystatin concentrations.

### 3.4. Microbial Fuel Cell Power

**Figure 7:**
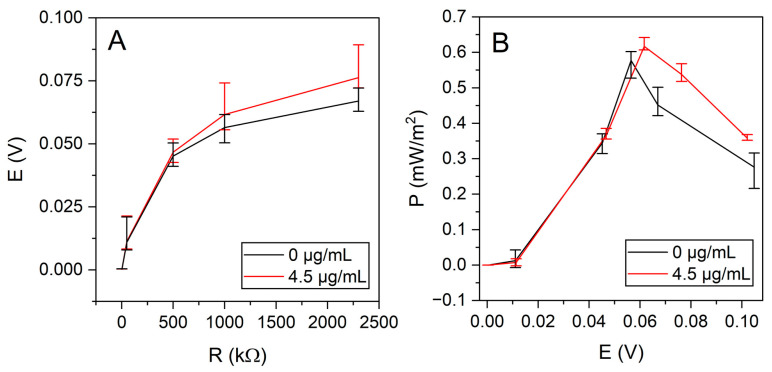
(**A**). Power versus potential, with untreated yeast (0 μg/mL nystatin) and nystatin-treated yeast (4.5 μg/mL nystatin). (**B**). Load dependence of potential with nystatin-treated (4.5 μg/mL) and untreated (0 μg/mL) yeast.

The power generated by the resulting MFC was determined by measuring the operating input using various loads (Figure 7).

The measured MFC potentials and power densities for untreated and nystatin-treated yeast under different external loads are summarized in Table 2.

The MFC generated 67.0 mV with untreated yeast and 76.3 mV with nystatin-treated yeast at 60 mM glucose under a 2.3 MΩ load. A maximum open circuit potential of 102 mV was achieved with untreated yeast and 104 mV with nystatin-treated yeast.

A yeast-based MFC producing a maximum power of 0.58 mW/m^2^ at 500 kΩ, generating 45.1 mV potential, was constructed using untreated yeast. Meanwhile, a power of 0.62 mW/m^2^ was achieved with nystatin-treated yeast at a load of 500 kΩ (Figure 7B).

This study focuses on the short-term electrochemical effects of nystatin treatment and mediator configuration; long-term operational stability will be investigated in future work.

The absolute power densities obtained in this study are low compared to those of larger or optimized microbial fuel cells; however, this is expected given the very small anode surface area (3 mm diameter), low biomass loading, and the mechanistic focus of this work. The electrode surface area, mass transport of mediators and substrates, the intrinsic metabolic rate of the yeast, and oxygen reduction kinetics at the cathode under passive aeration conditions primarily limit power output. Future improvements could be achieved by increasing electrode surface area, employing high-surface-area electrode materials, increasing cell density, optimizing mediator concentrations, and improving cathode catalysis or oxygen supply.

## 4. Conclusions

A key novel aspect of this work is the demonstration that controlled, sub-lethal biological membrane permeabilization can be used as an experimental handle to enhance electron transfer in MFCs. While previous studies have focused primarily on mediator efficiency or electrode modification, the present results show that modifying the biological interface itself—specifically the yeast membrane—can increase local electrochemical activity without compromising viability. Moreover, the use of SECM enables direct visualization of this enhancement at the microscale, providing mechanistic insight into how membrane permeability influences electron transfer. This approach introduces membrane permeability as a new, tunable design parameter in bioelectrochemical systems. This study demonstrates that controlled, sub-lethal permeabilization of the yeast cell membrane using nystatin can enhance mediator-assisted electron transfer in yeast-based MFCs.

Scanning electrochemical microscopy measurements reveal that the use of a dual-mediator system increases the local current response by approximately fivefold compared to a single mediator, and that nystatin-treated yeast exhibits higher local electrochemical activity than untreated yeast. Increasing the nystatin concentration further increased the maximum SECM current registered. MFC measurements show that nystatin treatment increases the maximum power density when compared with the same measurements using untreated yeast.

Taken together, these results indicate that the most effective configuration within the investigated parameter is a dual-mediator system combined with sub-lethal nystatin treatment during cultivation, which maximizes local electron transfer and power output while maintaining cell viability. These findings demonstrate that membrane permeabilization represents a tunable biological parameter for improving bioelectrochemical system performance.

## Figures and Tables

**Table 1:** Summary of experimental parameters and operating conditions.

Technique/Parameter	Experimental Condition	Purpose
**Viability assay**		
Nystatin concentration	4–5 µg/mL	Sub-lethal membrane permeabilization
Glucose concentration	60 mM	Metabolic substrate
Temperature	Room temperature	Physiological condition
**Cyclic voltammetry (CV)**		
Scan rate	0.1 V s^−1^	Redox peak resolution
Potential range	−0.6 to +0.8 V	Redox window
Potential step	0.01 V	Data resolution
Hydrophilic mediator	20 mM K_3_[Fe(CN)_6_]	Extracellular redox mediator
Lipophilic mediator	3 mM PQ (2 µL immobilized)	Intracellular electron shuttle
**Scanning electrochemical microscopy (SECM)**		
Tip radius	5 µm	Spatial resolution
Working distance	20 µm	Local current mapping
Tip potential	+400 mV	Generation-collection mode
Hydrophilic mediator	0.6 mM K_3_[Fe(CN)_6_]	Electron collection
Lipophilic mediator	0.04 mM PQ	Transmembrane electron transfer
**Microbial fuel cell (MFC)**		
Anode material	Graphite rod, 3 mm diameter	Working electrode
Yeast suspension volume	2 µL	Biocatalyst
Membrane pore size	3 µm	Cell retention
Cathode operation	Air-saturated electrolyte	Oxygen reduction
External load range	2.3 MΩ–100 Ω	Power characterization

**Table 2:** MFC potential and power density of untreated and nystatin-treated yeast under different external loads.

External Load	Voltage Untreated (mV)	Power Untreated (mW/m^2^)	Voltage Treated (mV)	Power Treated (mW/m^2^)
Open circuit	102	0	104	0
2.3 MΩ	67.0	0.28	76.3	0.36
1 MΩ	56.5	0.45	61.6	0.54
500 kΩ	45.1	0.58	46.7	0.62
50 kΩ	11.0	0.34	11.4	0.37

## Data Availability

The data presented in this study are available on request.
